# 24-h Potassium Excretion Is Associated with Components of the Metabolic Syndrome: Results from a National Survey Based on Urine Collection in Adults

**DOI:** 10.3390/nu13082689

**Published:** 2021-08-03

**Authors:** Assaf Buch, Rebecca Goldsmith, Lesley Nitsan, Miri Margaliot, Gabi Shefer, Yonit Marcus, Naftali Stern

**Affiliations:** 1 Tel Aviv-Sourasky Medical Center, Institute of Endocrinology, Metabolism and Hypertension, Tel-Aviv 64239, Israel; asafbu@tlvmc.gov.il (A.B.); mirim@tlvmc.gov.il (M.M.); gabish@tlvmc.gov.il (G.S.); yonitm@tlvmc.gov.il (Y.M.); 2 Nutrition Division, Ministry of Health, Jerusalem 9101002, Israel; gorebecca@gmail.com (R.G.); Lesley.Nitzan@moh.gov.il (L.N.); 3 The Sagol Center for Epigenetics of Aging and Metabolism, Institute of Endocrinology, Metabolism and Hypertension, Tel-Aviv 64239, Israel; 4 The Sackler Faculty of Medicine, Tel-Aviv University, Tel Aviv 69978, Israel

**Keywords:** potassium excretion, metabolic syndrome, sodium excretion

## Abstract

A balanced diet and weight loss are the first lines of treatment for the prevention of metabolic syndrome (MS). Dietary strategies may include changing the composition of macronutrients, adopting a particular dietary pattern as a Mediterranean diet. However, the role of micronutrients, particularly potassium, in the propensity for or treatment of the syndrome is unclear. The study aimed to examine the relationship between the presence of the MS and its risk factors and the 24-h potassium excretion as the most valid proxy for dietary intake. The analyses were performed as part of the national survey estimating sodium and other electrolytes excretion conducted between 2014–2016 in Israel. The survey included urine collection, anthropometric and blood pressure measurements, and a comprehensive medical questionnaire that included details on the intake of medications that may affect electrolyte secretion. A model was constructed to evaluate the probability for the MS. MS score and its probability were examined in relation to potassium excretion at different levels and in stratification to sex. A total of 581 participants were included in the analysis. The mean potassium excretion was 2818 ± 1417 mg. The prevalence of the MS was 18.5% among participants with above-average potassium excretion and about 10.4% among participants with lower-than-average excretion (*p* = 0.007). A dose–response relationship was observed between MS score and potassium: the higher the score, the lower was the excretion of potassium. Potassium excretion, rather than sodium excretion, correlated with all components of the MS and even predicted MS independently from other variables. This is the first study based on a national survey showing that potassium consumption, as represented by daily excretion in urine, is inversely related to the presence of MS components after adjustment for several leading variables and careful exclusion of participants taking drugs which may interfere in potassium excretion.

## 1. Introduction

Metabolic syndrome (MS) is a cluster of interconnected risk factors that are associated with increased risk of cardiovascular disease (CVD) and type 2 diabetes mellitus (T2DM). The risk factors include raised blood pressure, dyslipidemia (high triglycerides and low high-density lipoprotein cholesterol), raised fasting glucose, and central obesity [1,2]. Central obesity, weight gain, and/or high BMI are considered the key factors in developing metabolic syndrome, which induce or are at least linked to insulin resistance, low-grade inflammation in fat tissue, and ectopic fat deposition [3,4]. Nutritional contributing factors to the evolution of MS and its progression likely include excess energy intake with relatively high carbohydrate consumption and/or excess dietary saturated fatty acids [3,5]. Less attention was given to micronutrient intake and the MS. Abundant data supports the role of sodium intake with higher blood pressure (BP) [6,7,8], one of the five components of the MS score. Potassium intake alone or a part of a whole diet pattern was suggested as a possible protective factor against elevated BP [9,10]: not only can increased consumption lower blood pressure but may also provide direct vascular protection, even in the absence of concomitant reduction in arterial pressure per se. 

Consumption of dietary potassium in the general population in Western countries appears to be substantially lower than the Dietary Recommended Intake (DRI) of ≥4.7 g [11,12,13,14]. For example, in the National Health and Nutrition Examination Survey (NHANES) III, adults’ average daily potassium intake was 2.9–3.2 g for men and 2.1–2.3 g for women. Particularly impressive was the finding that only 10% of men and less than 1% of women consumed the proper DRI for potassium [11]. Two widely recommended diets entail an increase in the consumption of potassium: the Mediterranean diet and the DASH diet. In one study of the Mediterranean diet, better compliance with the diet was linked to higher consumption of dietary potassium as well as calcium and magnesium [15]. Higher than the average consumption of potassium (around the 75th percentile of the US consumption) is also an inherent feature of the DASH diet [16,17]. Whereas many clinical trials showed that high potassium intake, such as provided by the DASH diet is linked to lower blood pressure and may, indeed, lower blood pressure in interventional trials [18,19], the potential role of potassium intake in MS as a multi-component condition, beyond its likely effect on hypertension per se, has thus far generated little interest. Moreover, the few studies examining the association of potassium with the MS were limited to nutritional intake or did not consider the effect of potassium influencing drugs [20]. 

There are different methods to assess potassium intake based on the subjects’ own reports: 24-h dietary recall questionnaires, Food Frequency Questionnaires (FFQs), or dietary records. These methods present inherent methodological challenges as they are prone to report bias/inaccuracy. Not free of limitations, too, the measurement of the amount of potassium excreted in the urine in 24 h (24-h urinary potassium output) likely comprises the best available means to objectively assess potassium intake, assuming the gastrointestinal tract functions in a stable form during the collection. Under normal conditions, the majority of excess dietary potassium (~80–90%) is excreted by the kidneys to help maintain potassium homeostasis [21], and it is thus considered the gold standard for determining potassium intake [22,23]. However, some authorities maintain that urinary potassium represents a smaller fraction of ingested potassium due to more substantial K+ loss in the gut. Further, urinary potassium excretion is heavily affected by sodium delivery to the distal nephron, such that higher sodium intake elicits larger urinary potassium loss secondary to sodium/potassium exchange mechanisms in the kidney. 

In a post hoc analysis of a prospective trial, we have previously shown that an increase in dietary potassium consumption predicts the achieved reduction in BMI in a weight-loss diet program in obese patients with MS [24]. However, these post-hoc findings relied on subjective dietary self-reports, which may not necessarily represent potassium consumption [25,26]. In the present report we examined the potential association between potassium excretion in the urine and the MS components and likelihood, based on the first national representative sample of Israeli adults conducted in 2014–2016 [27]. We a priori hypothesized that higher potassium levels would be negatively associated with either the MS components or/and the presence of MS.

## 2. Materials and Methods

### 2.1. Population and Sampling

The Ministry of Health initiated the study as part of the National Lifestyle Improvement Program—”Efsharibari” (“Healthy is Possible”). One of the program’s goals is to achieve a reduction in sodium consumption. Therefore, the First Israeli National Sodium Survey was initiated so baseline levels of sodium as well as other electrolytes (such as potassium) can be evaluated before national intervention. The study took place at the institute of Endocrinology, Metabolism, and Hypertension (IEMH), Tel-Aviv Sourasky Medical Center (TASMC). This was a random sample composed of participants aged 20–65 years insured in three (out of four) Health Maintenance Organizations (HMOs–health funds). Due to locating problems, there is an under-representation of Arabs (6.3% compared to 14.2% for the same age groups in the population). The distribution of sex in the sample is similar to the distribution in the population (men 45.1% versus women 43.8%). There is under-representation of the 20–34 age group, and over-representation of the 55+ age group and over-representation of the Tel Aviv District [27].

The study was approved by each HMO’s own ethics committee (Helsinki) (approval had to be obtained from each committee separately), and signed informed consent was obtained from all participants. The sampling method was based on lists obtained from each of the three insurers and were divided into two age groups (25–44, 45–64) and two sex groups. More information on the original study and the sampling method can be seen elsewhere [27].

#### Assessment of Eligibility

(1) Inclusion criteria: people over the age of 20; people living in localities with a population of 20,000 or more; and people who speak Hebrew, Arabic, or Russian. (2) Exclusion criteria: people taking diuretics, angiotensin-converting enzyme inhibitors or angiotensin receptor blockers; people suffering from kidney failure; people suffering from heart failure, stroke, and liver disease; pregnant women; people hospitalized or living in institutions (not including sheltered housing); and people suffering from incontinence.

### 2.2. Study Flow and Protocol

The interviewers underwent three days of training in which all the survey contents were presented and practiced. In 2014, a pilot test was conducted that included examining the questionnaire and the anthropometric measurements planned for the study (no urine was collected at this point). The trained team made initial telephone contact with the respondents to obtain an agreement to participate and to be interviewed for the survey. The first interview was conducted at the interviewee’s home and included the delivery of the urine collection equipment and a detailed explanation of how the urine was to be collected. The second interview was also conducted at the interviewee’s home and included filling out a general questionnaire and nutrition questionnaires, weight and height measurements, measurements of blood pressure, and pulse. A urine sample was collected from the 24-h urine collection. The interviewee received a gift voucher according to that agreed upon.

### 2.3. Data Collection and Definition–Main Exposure Variables

#### 2.3.1. Potassium and Sodium Assessments

(a). Urine sample: The Ministry of Health adopted the protocol of the PAHO [22], which is the protocol of the World Health Organization for urine collection surveys to assess electrolyte intake. In the laboratory, the details of the sample were entered into a laboratory computer system, the identifying details were checked, and the samples frozen until the analyses were performed. Urinary creatinine, magnesium, sodium, potassium, calcium, protein, microalbumin, osmolarity, and specific gravity from the 24-h urine collection were measured by standard automated methods. Two-liter plastic containers for urine storage, urine cups, an insulated opaque carry bag, and disposable gloves were supplied. Urine collection started after the first morning urine void and continued for a full 24 h. The entire urine volume was collected and stored. The total urine volume was recorded. Whenever urine volume did not exceed 2 L, the container was first mixed and a sample then drawn by syringe and placed in a sterile urine collection cup. In cases where urine volume exceeded 2 L, thereby necessitating use of more than one container, a urine sample was drawn from each container in proportion to the volume in each bottle and then mixed.

(b). Dietary intake: Both potassium and sodium dietary intakes were estimated using a standardized 24-h dietary recall questionnaire (compliance was 99.7%) and the FFQ questionnaire (compliance was 95.1%) which was developed and validated by the Nutrition Division of the Ministry of Health and was previously used for the latest National Health and Nutrition Survey (Rav-Mabat survey) [28]. 

#### 2.3.2. A Standardized Questionnaire for Assessment of Other Explanatory Variables

A personal face-to-face interview was conducted in the interviewees’ homes using a structured questionnaire. The full questionnaire included demographic details, questions on health status (ever diagnosed with certain diseases), lifestyle as well as descriptive details addressing exercise habits, medication, and smoking. Information on age, sex, origin, country of birth, religion, level of religiosity, marital status, education, and income was also collected.

#### 2.3.3. Drug Therapy

In total, 284 (48%) reported that they were taking medications and/or nutritional supplements. Each subject was requested to bring/show the medications/supplements to the interviewer (compliance at this stage was 92.2%). Although we a priori excluded subjects taking drugs that affect sodium or potassium excretion, we reexamined the lists of drugs which emerged during the interviews with the interviewees and may have either been missed in the computerized data files or modified due to medical needs after the date that extraction from these files were carried out to ensure no administration of these drugs since the time of recruitment to the study to the time of the actual enrollment of each subject he or she could have been prescribed with these drugs by their physician. In total, we found 12 subjects who were taking ACE inhibitors. A sensitivity analysis was performed with and without their inclusion. 

### 2.4. Data Collection: Main Outcome—Metabolic Syndrome and Its Components

Following the interview, the interviewers measured blood pressure and heart rate using an electronic monitor—digital blood pressure monitor, A&D Instruments Ltd., Abingdon UK, model UA-767 (compliance was 94.2%), according to a protocol based on recommendations of the American Heart Association (AHA). Blood pressure and pulse were measured on the right arm, while sitting, and each measurement was carried out twice, with a minute’s rest between measurements. The final value was the average of two measurements which comprise the minimum repetitions for BP measurement as indicated by the High Blood Pressure Clinical Practice Guideline in 2017 [29]. Where there was a difference of 10% or more between measurements of either systolic or diastolic pressure, a third measurement was carried out and considered as the “true” measurement. 

Following blood pressure and pulse measurements, weight and height were also obtained, with compliance of 97.4%. According to a protocol, all measurements were carried out twice (and the average calculated) and included: standing height and weight. All measurements were carried out in light clothing without shoes. Weight measurements were carried out using a digital scale suitable for weighing 130 kg, with an accuracy of 0.5 kg.

Since the survey did not include blood testing, we built a likelihood model to estimate MS based on five components that were selected to approximate as much as possible the accepted and in consensus MS scoring system outlined by the ATP III guidelines [30]. MS likelihood was defined by the presence of three or more of the following criteria (*n*; % in sample): 1. The presence of diabetes (65; 11.2%); 2. Elevated blood pressure [≥130 mm for systolic (195; 33.5%); or ≥85 mm for diastolic (237; 40.8%)] or prescribed antihypertensive drugs not affecting potassium balance, with normotensive values (*n* = 4) (293; 50.4%); 3. Report on hypertriglyceridemia or prescribed drugs for hypertriglyceridemia (100; 17.2%); 4. Overweight/obesity (indicated by BMI ≥ 28.5 kg/m^2^) as a close proxy for high waist circumference [31]. Verified also by the first national nutrition and health survey for adults (MABAT)—81% of the participants with this BMI had abdominal obesity (data not shown) (189; 32.5%); and 5. Since we did not have direct measurement of HDL-C we included the diagnosis of fatty liver which was previously suggested as a potential indicator of MS [32] (64; 11.0%). In support of the inclusion of fatty liver in our model, in the absence of HDLc levels we: (a) found a positive correlation of diabetes with both fatty liver and hypertriglyceridemia in our sample (r = 0.266, *p* < 0.0001; r = 0.294, *p* < 0.0001, respectively); and (b) calculated that 51% (*n* = 33) of the subjects with a BMI ≥ 30 kg/m^2^ who reported to have fatty liver had also reported hypertriglyceridemia and that 58% (*n* = 37) of the subjects with fatty liver had a BMI ≥ 30 kg/m^2^. Each MS criterion’s presence was considered one point summing up for a score between “0” to “5”. 

### 2.5. Data Handling and Analysis 

Urine tests were recorded in an Excel file at the laboratory and this file was transferred to the Ministry of Health. The general questionnaires were entered in the SPSS program. In addition, FFQ nutrition questionnaires were entered, and dietary frequency data converted to daily intake. The 24-h dietary recall questionnaires were entered by a dietitian familiar with the “Tzameret 3” dietary software, a program developed by the Ministry of Health to process nutrition questionnaires [33]. “Tzameret” enables recording of food intake and calculation of nutrients intake and comparison with the recommendations. The software uses the nutrient data in the BINAT nutrient database, maintained and updated by the Nutrition Division of the Ministry of Health. When data entry of the dietary recalls was completed, Excel files were extracted with summary data, and these files were merged with the other data files. Data analysis was performed with SPSS software (24.0; IBM). All continuous variables are presented as mean ± SD and categorical or numerical variables as proportion. When caloric intake was included in the analyses, we excluded outliers with an intake of ≥5500 (*n* = 3) and <800 kcal/day (*n* = 22). 

We found all continuous variables to follow a normal distribution. A univariate analysis was conducted to test the difference between the potassium groups (stratified based on the mean values): (1) high potassium excretion vs. low potassium excretion (using a t-test); and (2) the associations between the different MS-likelihood scores and potassium excretion (tested by one-way ANOVA). We also analyzed the difference between MS-likelihood groups where the sample was stratified into high vs. low likelihood for the MS (a t-test was performed). To test the potential collinearity between variables as well as their correlation, we used the Spearman or the Pearson correlations (for categorical or continuous variables, respectively). Considering any between-confounder correlation > 0.7 as indicative of co-linearity [34], none was found: the highest derived correlation was r = 0.382. Using multivariate linear regressions, we examined the relation between electrolytes excretion (potassium or sodium) and MS-likelihood score, adjusted for several potential confounders (e.g., age, sex, and others as detailed in the results). These confounders were selected based on existing literature and their distribution in the stratified exposure variables (high potassium vs. low potassium excretion). Lastly, we performed several sensitivity analyses to ensure the internal validity of our findings where we repeated the main analyses with the exclusion of the following: (1) subjects taking drugs that might affect potassium excretion in the urine (*n* = 11); (2) subjects taking any “metabolically-related drugs” classified as anti-platelet (*n* = 28), lipid-lowering (*n* = 39; mainly statins–90%), diabetes (*n* = 17) and BP drugs (*n* = 34) (total including duplicates *n* = 65); and (3) subjects reporting on a non-normal activity level on the day of urine collection (*n* = 162). To rule out the possible contribution of hypertension/increased blood pressure on the potassium–MS association, we performed an additional sensitivity analysis in which we excluded elevated blood pressure from the MS-likelihood model (*n* = 293). Then we reexamined the association of potassium excretion with MS score (scores range 0–4).

## 3. Results

### 3.1. Subjects’ General and Lifestyle Characteristics and Electrolytes Levels

Out of 594 subjects in the original survey, the analysis included 581 subjects with a complete potassium urine sample. The mean age was 46.3 ± 12.1 years, 54.2% of the sample were female, 74.2% were married (or declared living with a partner), and 22.2% identified themselves as current smokers (Table 1). 

The mean 24 h excretion of potassium and sodium was 2818 ± 1417 and 3826 ± 1691 mg/day, respectively (mean sodium–potassium excretion ratio was 1.5 ± 0.64). Adjusted to 24-h creatinine excretion (gram), the mean levels were 2270 ± 93 for potassium and 3130 ± 138 mg for sodium (Table 1). Below mean levels of potassium excretion [defined as 24 h potassium excretion (mg) adjusted to 24-h creatinine excretion < 2270 mg/gram] was found in 56% of the participants. Marital status, educational level, and socioeconomic status did not differ significantly across potassium excretion groups.

### 3.2. Subjects’ Health and Metabolic Characteristics

The mean MS score was 1.21 ± 1.18, which was contributed to by the rates of elevated blood pressure (50%), overweight/obesity (33%), hypertriglyceridemia (17%), diabetes (11%), and fatty liver (11%). According to the a priori selection criteria, 87 (18.5%) of the subjects were categorized with high likelihood for MS (as expressed by MS score ≥ 3), of whom 57 subjects had a score of “3”, 24 were classified having a score of “4”, and only 6 subjects were classified as having all MS criteria (a score of “5”). Mean measured blood pressure was 125 ± 18.57 for systolic blood pressure and 80.96 ± 11.81 for diastolic blood pressure. The percentage of subjects treated with medication indicative of components of the metabolic diagnoses (diabetes, blood pressure, and dyslipidemia) or with anti-platelet drugs was relatively low (<7%) (Table 2). 

### 3.3. Associations of Potassium Excretion with the Metabolic Syndrome Components

**Figure 1:**
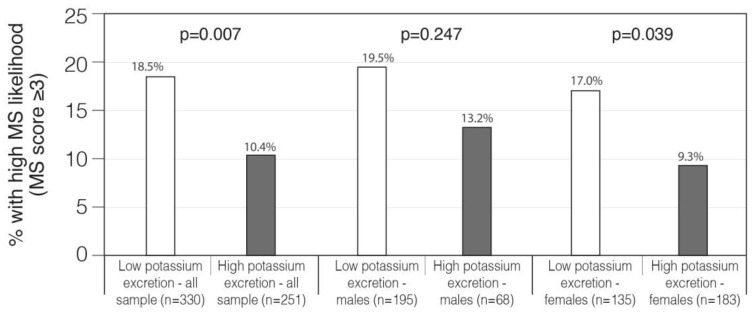
MS-likelihood prevalence according to potassium excretion levels in the total sample and gender-stratified. 24-h potassium excretion was normalized for 24 h creatinine excretion (mg). Potassium exposure levels are based on mean levels (≥2.27 was defined as high potassium excretion vs. <2.27 defined as low potassium excretion). MS-likelihood rates were compared using Chi-square-test.

When potassium excretion levels (adjusted to creatinine) were clustered into high vs. low levels (based on mean excretion levels), a negative association was found with mean MS score. This negative association indicated that the prevalence of MS likelihood was lower in subjects with higher than the mean potassium excretion as compared to subjects with lower than the mean potassium excretion (10.4% vs. 18.5%, *p* = 0.007). Sex modified the association between “MS-likelihood prevalence” and potassium excretion, as a significant “effect” was found in females only (*p* = 0.039) (Figure 1). Significant associations between single MS components and potassium excretion levels (above compared to below average) were found for overweight/obese (BMI ≥ 28.5 kg/m^2^) (24% compared to 39%), elevated blood pressure (≥130 mm for systolic or ≥85 mm for diastolic) (46.9% vs. 56.5%) and with fatty liver (7.5% vs. 13.5%) in the high vs. low potassium excretion groups, respectively (Table 3).

**Figure 2:**
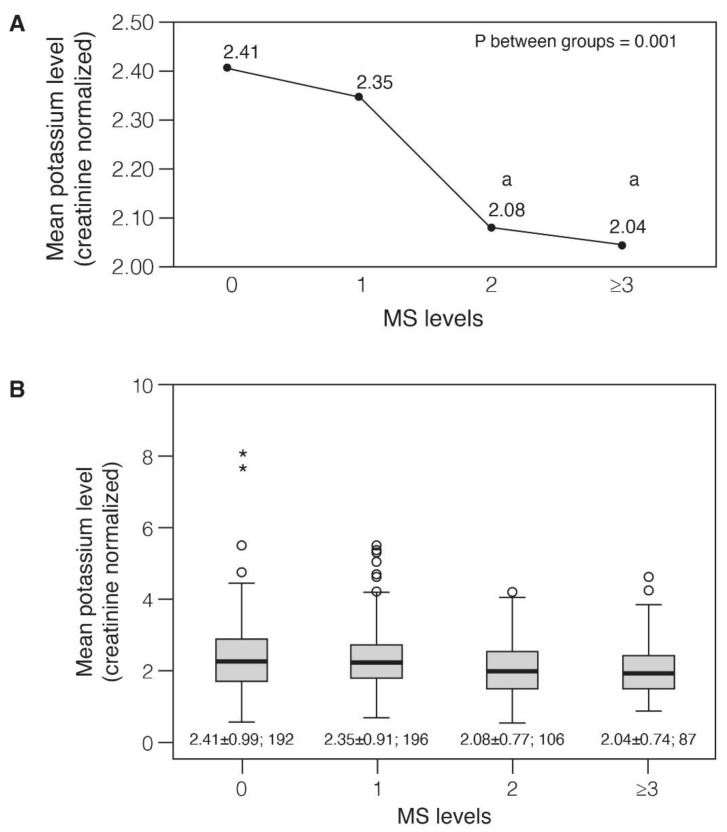
Mean potassium level as a function of MS levels. (**A**), Mean potassium levels according to MS levels; (**B**), Mean potassium levels and distribution according to MS levels – presented by BOX plot. Difference between groups was examined by one-way ANOVA test; Scheffe correction for multiple comparisons was implemented to test the difference between each group. “a” mean levels are significantly different than group “0” (no MS likelihood) (** *p* < 0.05). Abbreviations: MS, Metabolic syndrome.

As shown in Figure 2 groups of MS (represented by MS scores) were compared based on their potassium excretion levels using one-way ANOVA test (Figure 2A) and box plots (Figure 2B). Mean potassium excretion levels (creatinine normalized) declined with higher calculated MS-likelihood scores (*p* between groups = 0.001) from 2.41 ± 0.99 among subjects with no MS criteria to 2.04 ± 0.74 among individuals with high-likelihood for MS (score ≥ 3) (*p* < 0.05; Figure 2A,B). There was higher variability in potassium excretion in groups representing low MS-likelihood (scores = 0, 1, 2) than the group representing higher likelihood (MS score ≥ 3). Stratified by gender, the mean observed potassium excretion levels were inversely declining as the calculated MS-likelihood score increased, however, did not reach significance (data not shown). The exclusion of elevated blood pressure (≥130 mm for systolic or ≥85 mm for diastolic) from the MS model did not alter the association between potassium excretion and MS. Among normotensive subjects with high potassium excretion, the mean MS-likelihood score was 0.54 ± 0.96 compared to a score of 0.75 ± 1.1 in subjects with low excretion levels (*p* = 0.016). 

### 3.4. The Relation of Potassium and Sodium Excretion and Intake with Other Metabolic Syndrome Variables

As indicated by Table 3, there was a low, yet significant, negative correlation between potassium excretion (adjusted to creatinine) and MS-likelihood score (r = −0.161, *p* < 0.01). A closer look on the specific correlations between potassium excretion levels and MS components revealed negative correlations with the following: (1) BMI (as a continuous variable) and overweight/obesity (as a dichotomous variable) (r = −0.142 and r spearma*n* = −0.162, *p* < 0.01); (2) measured elevated blood pressure (dichotomous) and with systolic and diastolic levels (r spearma*n* = −0.108, *p* < 0.01; r =−0.095 and r = −0.143, *p* < 0.05); and (3) fatty liver diagnosis (dichotomous) (r spearma*n* = −0.132, *p* < 0.01). In contrast, sodium excretion did not correlate with any of the MS components with the exception of positive correlations with mean systolic blood pressure and elevated measured blood pressure (among females). Also, none of these variables was related to potassium intake as assessed by food recall or FFQ (Table 3). However, sodium to potassium excretion ratio was positively correlated with MS-likelihood score and MS components as follows: obesity (r = 0.157, *p* < 0.01) or BMI (r = 0.151, *p* < 0.01), elevated blood pressure (r = 0.113, *p* < 0.01), and fatty liver (r = 0.100, *p* < 0.05) (Table 3). 

### 3.5. The Contribution of Food Groups to the Potassium Intake as Reported in 24-h Recalls

**Figure 3:**
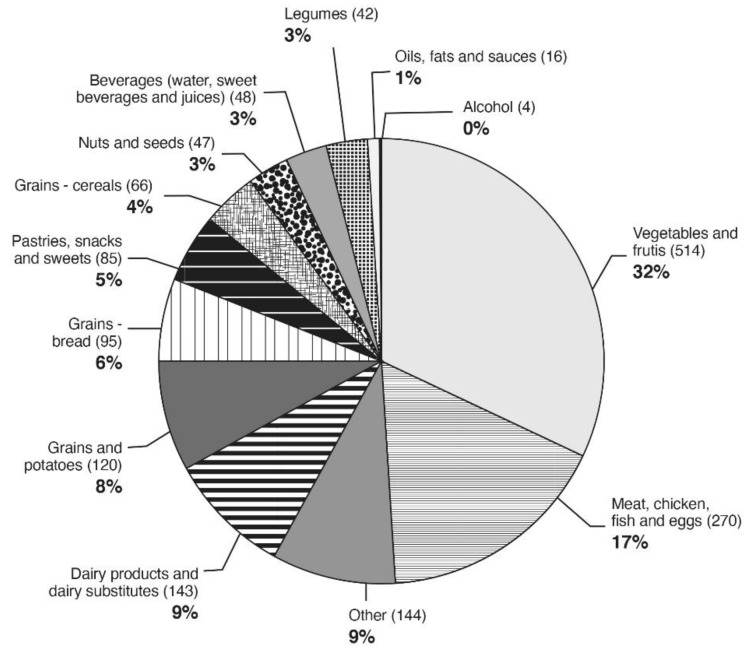
Food groups’ contribution to potassium intake as assessed by 24-h food recall.

The main food groups contributing to potassium intake (therefore, for excretion) were: vegetables and fruits (mean intake of 514 g per day; 32% of the total intake), followed by the consumption of non-dairy protein (fish, meat, chicken, and eggs) which accounted for 17% of the total potassium intake. Dairy products and their substitutes (soy beverages, for instance) contributed 9% of the total intake, followed by grains other than bread and potatoes, and grains from bread (8% and 6%, respectively) (Figure 3). 

### 3.6. Multivariate Analyses 

The independent correlations between MS-likelihood score and creatinine adjusted potassium, sodium excretion, and sodium potassium ratio are summarized in Table 4. As reflected by the regression models, potassium excretion had a stronger negative correlation with MS-likelihood score than the correlation of sodium/potassium excretion ratio with MS score (standardized coefficient for potassium excretion/beta [B], −0.222 vs. B for sodium/potassium excretion ratio, 0.140; data not shown), whereas sodium excretion did not independently correlate with MS-likelihood score. Furthermore, the relative attributed variance in the MS-likelihood score explained by potassium excretion was the highest compared to the other models (R square change = 4.8%) (Table 4). Each ratio-increment of 1 in potassium/creatinine excretion, predicted a decrease of −0.285 in the calculated MS-likelihood score. These inverse potassium–MS associations were independent of other explanatory variables such as age, sex, physical activity, and caloric intake (Table 4). The results were not altered by the type of method of the regression (similar results for an “enter” method vs. the current stepwise method; data not shown). 

Sensitivity analyses were performed each time with the exclusion of certain factors and suggested similar results (data not shown). In one analysis subjects taking drugs that might have an effect on potassium excretion were excluded (*n* = 11), no change occurred in this step (B = −0.220; 95% CI, −0.322, and −0.117) with a slightly lower R square of the model (13.6%). The same effect was seen for sodium/potassium excretion ratio (B = 0.233; 95% CI, 0.094, and 0.372). Further sensitivity analysis excluded all subjects taking drugs with “metabolic effect” (lipid, glucose, BP lowering and anticoagulant drugs) (*n* = 65). The results also remained consistent (B = −0.218; 95% CI, −0.318, and −0.118 for potassium excretion and B = 0.200; 95% CI, 0.064, and 0.336 for sodium-potassium excretion ratio). The last sensitivity analysis was performed with the exclusion of all subjects who reported a non-normal activity level on urine collection day (*n* = 164). This analysis strengthened the quality of the prediction model for both potassium excretion and sodium–potassium excretion ratio (R square of 17.2% and 16.1%, respectively). Both potassium excretion and sodium/potassium excretion ratio still independently predicted the change in MS-likelihood score (B−0.217; 95% CI, −0.337, −0.096 and B = 0.232; 95% CI, 0.060, and 0.404). Sodium excretion did not correlate with MS-likelihood score in all sensitivity analyses. 

**Figure 4:**
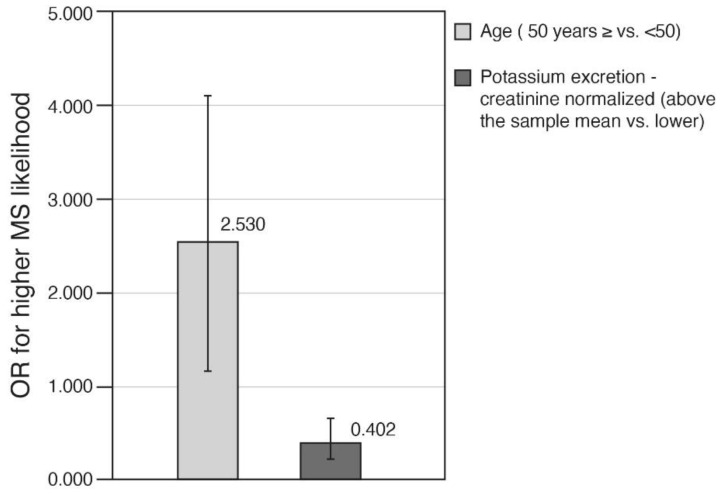
Adjusted Odds Ratios for high MS likelihood explained by potassium excretion and other explanatory variables. ORs using stepwise logistic regression predicting MS likelihood (=1 as compared to lower likelihood, =0) by potassium excretion (normalized to creatinine levels) –adjusted to the potential confounders: age (≥50 vs. <50 years), sex (0—female, 1—male), physical activity (at least 20 min/wk.: 0—no, 1—yes) and caloric intake (Kcals). Caloric intake was entered excluding outliers (*n* = 25) (as mentioned in the methods section). Only significant variables are presented by the bars; error bars represent 95% confidence intervals. Model summary by Nagelkerke R Square, 8.5%. Abbreviations: MS, Metabolic syndrome; OR, Odds Ratio.

Finally, in a multivariate logistic regression, the odds for high MS-likelihood score (a score ≥ 3 vs. <3) were 0.402 (60%) lower in those with high vs. low potassium excretion (≥mean level vs. <mean levels) (stepwise regression adjusted for age, physical activity, sex, and caloric intake) (Figure 4).

## 4. Discussion

The present study assessed for the first time the association between 24-h potassium excretion as compared to potassium intake, sodium excretion, and MS likelihood and its components in a representative sample of adult Israelis. Several new findings emerged from our national survey: (1) 24-h potassium excretion (normalized to creatinine) was inversely associated with MS likelihood and significantly correlated with its specific components, independent of age, sex, physical activity, and caloric intake; (2) potassium excretion, but not sodium excretion, was a significant predictor of MS likelihood and its components and this association was not weakened by the exclusion of the well-recognized potassium or sodium relation with elevated blood pressure from the calculation; (3) whereas the 24-h potassium excretion was associated with MS and its components, this linkage could not be found between MS and potassium intake as assessed by either 24-h recall or FFQ, likely reflecting the limitations of dietary questionnaires relative to actual measurements. 

In the past few years several reports, mainly from Eastern Asia have described the possible linkage of potassium excretion and MS likelihood based on cross-sectional analyses [20,35,36,37]. However, reflected by our MS-likelihood model, to our knowledge, this is the first report suggesting a possible “role” of potassium in the MS likelihood assessed by 24-h potassium excretion. In contrast to our study, a recent cross-sectional analysis conducted in 1906 Chinese participants showed that the MS was not related to potassium excretion, but rather to sodium excretion [36] or sodium to potassium excretion ratio [35]. In a recent meta-analysis based on cross-sectional studies, MS prevalence was estimated at 20% and inversely associated with potassium intake (*n* = 3 studies) or serum levels, (*n* = 1), with a pooled OR of 0.75 (95% CI: 0.50–0.97)] [20]. Interestingly, we have shown that lower potassium excretion is associated with MS prevalence and was independent of the association between potassium and blood pressure, as there was a univariate relation with other MS components. Lower potassium and sodium to potassium excretion ratio in our cohort were associated with obesity and fatty liver. In one Chinese study, sodium/potassium excretion ratio was found to be associated with central obesity [35]; however, this was attributed to sodium rather than potassium excretion [36]. 

The dietary food groups from which potassium intake mainly derived in the present report were vegetables and fruits, followed by poultry and fish, dairy products, and grains (Figure 3). The consumption of these potassium-containing foods is in accord with the DASH dietary guidelines to treat hypertension [38], one of the MS components. The inverse and caloric independent association of potassium excretion (as a better proxy for intake) with MS found in the current study may legitimize such dietary consumption with respect to MS prevention. However, further prognostic studies relating potassium excretion with its intake and with MS incidence are needed to confirm this hypothesis-generating observation. 

The current study has several limitations. The first is the model we rely on, a post hoc approximation but not a precise application of the well-defined MS components. As such there may be underrepresentation of people with abdominal obesity (BMI ≥ 30 kg/m^2^ was chosen as a proxy of central obesity). Moreover, with the inclusion of diabetes in the components of MS, but not impaired glucose levels, prediabetic subjects are not represented. This likely explains the relatively low prevalence of MS likelihood in our study (≈11%) as the prevalence of subjects with impaired fasting glucose in obesity is sizable. Insulin per se can affect urinary potassium excretion via multiple mechanisms [39]. However, the sensitivity analysis we performed excluding all subjects taking any medications with “metabolic effect”, including diabetes medications (*n* = 17), showed that our findings were not appreciably affected. The results in our analysis, as in any observational study, could be attributed to residual confounding. The limited explained 15% variance in MS predicted by potassium excretion adjusted to other variables is consistent with the concept that other essential factors are related to the MS. 

The advantages of our study include the relatively high internal validity of our methodology contributed by the following: (1) reliance on 24-h excretion [unlike most studies on the topic reporting intake [20,37,40,41,42]]; (2) the rigorous exclusion of participants prescribed with medications possibly interfering potassium flux in the body; and (3) several sensitivity analyses supporting the observed role of potassium in MS likelihood. Regarding external validity, to the best of our knowledge, it is also the first study performed in a non-Asian population where nutritional intake is closer to a westernized pattern. 

## 5. Conclusions

This is the first study to show on a national level that 24-h potassium excretion was associated with MS likelihood and its components, including obesity, elevated blood pressure, and fatty liver. Lower potassium excretion was linked to a higher MS-likelihood score, independent of age, sex, physical activity, and caloric intake. These findings were not replicated for sodium excretion or dietary potassium intake. 

## Figures and Tables

**Table 1:** Participants’ general and lifestyle characteristics with stratification to potassium excretion groups (exposure).

	Total Sample (*n* = 581)			High Potassium Excretion (Creatinine Adjusted) ^1^ (*n* = 254)			Low Potassium Excretion (Creatinine Adjusted) ^1^ (*n* = 327)		
	All	Females (*n* = 318)	Males (*n* = 263)	All	Females (*n* = 185)	Males (*n* = 69)	All	Females (*n* = 133)	Males (*n* = 194)
Age (years)	46.27 ± 12.14	46.27 ± 11.61	46.36 ± 12.76	48.23 ± 11.74 **	47.23 ± 11.51	50.90 ± 12.01 **	44.82 ± 12.24	44.92 ± 11.65	44.74 ± 12.66
Gender (% females)	54.7	---	----	72.8 **	---	----	40.7	-----	----
Education (years)	14.74 ± 3.15	14.88 ± 11.61	14.63 ± 3.08	14.95 ± 3.14	15.13 ± 3.31	14.68 ± 3.38	14.5 ± 2.91	14.53 ± 2.87	14.68 ± 3.38
Marital status (% married/ living with a partner) ^2^	74.2	73.6	74.9	76.8	75.7	79.7	72.2	70.7	73.2
Socioeconomic status ^3^ (% defined as high)	28.1	18.3	39.5	26.2	17.4	49.1	29.5	19.6	36.2
Socioeconomic status ^3^ (% defined as low)	32.6	42.9	20.5	35.6	43.5	15.1	30.3	42.2	22.4
Ethnicity/religion (% Jews and others [non-Arab Christians]) ^4^	94.7	97.2	91.6	97.6 **	97.8	97.1	92.4	96.2	89.7
Smoking ^5^ (% current smokers)	22.2	20.1	24.7	17.7	18.4	15.9	25.7	22.6	27.8
Smoking ^5^ (% past smokers)	17.4	14.5	20.9	18.5	16.8	23.2	16.5	11.3	20.1
Any physical activity, at least 20 min, a week	52.0	51.9	52.1	57.9 *	58.9 **	55.1	47.4	42.1	51.0
All activity, how often ^6^ (% performing at least three times a week)	31.8	30.5	33.4	37	36.2	39	28	22.5	31.4
% participating in intense physical activity in a week	34.4	28.0	42.2	35.0	31.4	44.9	33.9	23.3	41.2
Energy intake by 24 h recall (Kcals) (*n* = 567)	1925.27 ± 764.35	1678.72 ± 599.77	2214.65 ± 840.96	1803.11 ± 682.81 **	1678.41 ± 580.44	1925.27 ± 764.35	2095.75 ± 836.71	1679.17 ± 628.73	2257.4778 ± 840.60

Notes: Data is shown as Mean ± SD for continuous variables (range) or % for nominal/categorical variables. Significance: males vs. males, females vs. females, and all vs. all between groups (high vs. low excretion groups) significantly different (* *p* < 0.05); or ** for *p* < 0.01. ^1^ Adjusted for 24 h creatinine excretion (mg). Potassium exposure levels are based on mean levels (≥2.27 was defined as high potassium excretion vs. <2.27 defined as low potassium excretion); ^2^ Assessed as: 1 = Single, 2 = Married/living with a partner, 3 = Divorced/separated, 4 = Widowed, and 5 = Other; ^3^ Categorized into high, medium or low socioeconomic status; ^4^ Originally categorized into: 1 = Jewish, 2 = Christian (not Arab), 3 = Arab Muslim, 4 = Arab Christian, 5 = Druze, and 6 = Other. Dichotomized into: 1 = Jews and others, 2 = Arabs; ^5^ Assessed as current, previous, or never smoker; ^6^ Among those reported performing physical activity: 1 = 5 times a week or more, 2 = 4 times/week, 3 = 3 times/week, 4 = 1–2 times/week, and 5 = less than once/week; ^7^ Excluding missing values (*n* = 2) and outliers (intake > 5500 kcals or intake < 800 kcals; *n* = 25); χ^2^ test for categorical variables and *T*-test (or Mann–Whitney) for continuous variables. Abbreviations: BMI, body mass index; MS, Metabolic Syndrome.

**Table 2:** Participants’ health/metabolic characteristics with stratification to potassium excretion groups (exposure).

	Total Sample (*n* = 581)			High Potassium Excretion (Creatinine Adjusted) ^1^ (*n*= 254)			Low Potassium Excretion (Creatinine Adjusted) ^1^ (*n*= 327)		
	All	Females (*n* = 318)	Males (*n* = 263)	All	Females (*n* = 185)	Males (*n* = 69)	All	Females (*n* = 133)	Males (*n* = 194)
24 h urine volume (mL/day)	1800.88 ± 840.82	1827.75 ± 872.66	1756.16 ± 769.86	2033.50 ± 849.03	2023.19 ± 850.93 **	2061.16 ± 849.50 **	1610.35 ± 762.32	1555.90 ± 831.57	1647.68 ± 710.79
24 h sodium excretion (creatinine adjusted)	3.13 ± 1.38 (0.3–14.37)	3.34 ± 1.27 (0.86–10.32)	2.88 ± 1.46 (0.3–14.77)	3.58 ± 1.65 **	3.61 ± 1.35 **	3.50 ± 2.29 **	2.78 ± 0.99	2.96 ± 1.04	2.66 ± 0.93
Sodium/potassium excretion ratio	1.50 ± 0.64 (0.13–4.96)	1.41 ± 0.56 (0.36–3.58)	1.62 ± 0.72 (0.13–4.96)	1.20 ± 0.52	1.20 ± 0.44 **	3.50 ± 2.29 **	1.74 ± 0.63	1.70 ± 0.57	2.66 ± 0.93
BMI (kg/m^2^) (*n* = 579) (range)	26.78 ± 5.08 (13.77–52.4)	26.55 ± 5.50 (13.77–52.40)	27.17 ± 4.56 (16.82–49.35)	26.01 ± 4.87 **	25.76 ± 5.17 **	26.65 ± 3.94	27.47 ± 5.18	27.63 ± 5.76	27.36 ± 4.76
%with BMI ≥ 28.5 kg/m^2^ (*n* = 566)	32.5 (189)	32 (101)	33 (88)	24 (60) **	24 (45) **	22 (15) *	39 (129)	42 (56)	37 (73)
% defined with elevated blood pressure according to MS criteria ^2^	52.3	42.4 (129)	64.6 (157)	46.9 *	40.9	63.1%	56.5	44.5	65.2%
Systolic blood pressure (mm/Hg) (range)	125.02 ± 18.57 (86–198)	120.50 ± 17.99 (86.00–180)	130.47 ± 17.42 (98.50–198)	122.24 ± 17.35	119.99 ± 17.06	128.36 ± 16.77	127.05 ± 18.95	121.21 ± 19.25	131.24 ± 17.64
Diastolic blood pressure (mm/Hg) (range)	80.96 ± 11.81 (48–136)	78.15 ± 11.17 (48–136)	84.46 ± 11.79 (58.00–133.00)	79.09 ± 11.27	77.92 ± 11.14	82.25 ± 11.08	82.42 ± 12.12	78.47 ± 11.25	85.27 ± 11.96
% treated with blood pressure drugs	5.9 (34)	5 (16)	6.8 (18)	7.1 (18)	5.9	10.1	4.9 (16)	3.8	5.7
% reporting diabetes (*n*)	11 (64)	9.4 (30)	12.9 (34)	10.6 (27)	8.6	15.9	11.3 (37)	10.5	11.9
% treated with diabetes drugs (*n*)	2.9 (17)	1.3 (4)	4.9 (13)	3.5 (9)	1.6	8.7	2.4 (8)	0.8	3.6
% reporting fatty liver	10.8 (63)	7.9 (25)	14.4 (38)	7.5 (19) *	5.9	11.6	13.5 (44)	10.5	15.5
% reporting diagnosis of hypertriglyceridemia	17.0 (99)	16.4 (52)	17.9 (47)	15.4 (39)	14.6	17.4	18.3 (60)	18.8	18.0
% treated with lipid- lowering drugs (*n*)	6.7 (39)	5.7 (18)	8 (21)	7.9 (20)	5.4	14.5 *	5.8 (19)	6.0	5.7
% treated with anti- platelet drugs (*n*)	4.8 (28)	2.5 (8)	7.6 (20)	5.1 (13)	2.7	11.6	4.6 (15)	2.3	6.2
Mean MS score	1.21 ± 1.18	1.07 ± 1.16	1.39 ± 1.19	1.02 ± 1.11 **	0.94 ± 1.07 *	1.26 ± 1.16	1.36 ± 1.22	1.25 ± 1.25	1.44 ± 1.20
% with high likelihood for MS (*n*)	15 (87)	12.6 (40)	18 (47)	10.4 (26)	9.3 (17)	13.2 (9)	18.5 (61)	17 (23)	19.5 (38)

Notes: Data is shown as Mean ± SD for continuous variables (range) or % for nominal/categorical variables. Significance: males vs. males, females vs. females, and all vs. all between groups (high vs. low excretion groups) significantly different (* *p* < 0.05); or ** for *p* < 0.01. ^1^ Adjusted for 24 h creatinine excretion (mg). Potassium exposure levels are based on mean levels (≥2.27 as defined as high potassium excretion vs. <2.27 defined as low potassium excretion); ^2^ ≥130 mm for systolic or ≥85 mm for diastolic (based on the average of two measurements according to a standardized protocol); χ^2^ test for categorical variables and *t*-test (or Mann–Whitney) for continuous variables. Abbreviations: BMI, body mass index; MS, Metabolic Syndrome.

**Table 3:** Pearson and Spearman correlation coefficients of electrolytes variables and other potential explanatory variables for the metabolic syndrome as well as its components.

	Potassium Excretion (Creatinine Adjusted) (*n* = 581)	Potassium intake by 24-h ReCall (Adjusted for Energy) (*n* = 556)	Potassium Intake by FFQ (Adjusted for Energy) (*n* = 510)	Sodium/Potassium Excretion Ratio (*n* = 580)	Sodium Excretion (Creatinine Adjusted) (*n* = 580)
Age (years)	0.184 ** (M: 0.174 **) (F: 0.217 **)	0.212 **	0.086	−0.125 ** (M: −0.176 **)	0.068 (F: 0.122 *)
Caloric intake (Kcals)	−0.164 **	NR	NR	0.129 **	−0.037
BMI (kg/m^2^)	−0.142 ** (F: −0.142 *)	0.017	−0.055	0.151 ** (F: 0.216 **)	0.022
Mean systolic blood pressure (mm/Hg)	−0.095 *	0.028	0.02	0.095 *	0.018 (F: 0.136 *)
Mean diastolic blood pressure (mm/Hg)	−0.143 ** (M: −0.186 **)	−0.031	−0.02	0.095 *	−0.016
Elevated blood pressure-measured (yes/no)	−0.108 *	0.073 (F: 0.131 *)	0.021	0.113 **	0.037 (F: 0.118 *)
All physical activity frequency ^1^	−0.113*	−0.078	−0.219 ** (M: −0.227 *) (F: −0.247 **)	0.065	−0.027
Fatty liver (yes/no)	−0.132 **	−0.05	−0.210 ** (M: −0.214 **) (F: −0.131 *)	0.100 *	−0.022
Diabetes (yes/no)	−0.001	−0.03	0.023	0.048	0.051
Overweight/obese (BMI ≥ 28.5 kg/m^2^) (yes/no)	−0.162 **	0.029	−0.062	0.157 **	0.018
Hypertriglyceridemia (yes/no)	0.003	0.052	−0.022	−0.059	−0.042
MS score	−0.161 ** (M: −0.127 *) (F: −0.119 *)	−0.055	−0.051	0.119 ** (F: 0.126 *)	−0.018

Notes: Correlation coefficients using Pearson (for continuous variables) or Spearman’s rho (for categorical variables). * Correlation is significant at the 0.05 level (two-tailed); ** Correlation is significant at the 0.01 level (two-tailed); correlations for gender added only when significant. Gray background indicates significant results in the level of the whole sample. ^1^ Among those reported performing physical activity: 1 = 5 times a week or more, 2 = 4 times/week, 3 = 3 times/week, 4 = 1–2 times/week, and 5 = less than once/week; Abbreviations: BMI, body mass index; F, Females; FFQ, Food Frequency Questionnaire; M, males; NR, Not Relevant.

**Table 4:** Adjusted linear correlations between electrolytes measurements and metabolic syndrome score.

	Model Including Potassium Excretion (Creatinine Adjusted) (*n* = 554)	Model Including Sodium/Potassium Excretion Ratio (*n* = 554)	Model Including Sodium Excretion (Creatinine Adjusted) (*n* = 554)
Variables included in the model (order in regression/level of “impact”)	(1) Age; and (2) potassium excretion	(1) Age; ( 2) Sodium/potassium excretion ratio; and (3) Sex	(1) Age; (2) Sex; and (3) Physical activity
Variables excluded from the model	Caloric intake; Sex; Physical activity	Caloric intake; Physical activity	Sodium excretion; Caloric intake
Coefficients for each variable (95% CI)	Age, 0.032 (0.025, 0.04); Potassium excretion, (−0.285–0.387, −0.183)	Age, 0.030 (0.022, 0.038); Sodium/potassium ratio, 0.255 (0.109, 0.402); and Sex, 0.275 (0.087, 0.462)	Age, 0.029 (0.021, 0.037); Sex, 0.326 (0.140, 0.512); and Physical activity, −0.201 (−0.387, −0.015)
Total R square of the model (% related to the electrolytes excretion)	13.4 (4.8)	12.4 (2.5)	11.3 (NI)

Notes: Correlations coefficients using stepwise linear regression predicting MS score by either potassium excretion, sodium excretion (each adjusted to creatinine levels), or sodium/potassium ratio—all adjusted to the potential confounders: age (years), sex (0—female, 1—male), physical activity (at least 20 min/wk.: 0—no. 1—yes); and caloric intake (Kcals). Caloric intake was entered excluding outliers (*n* = 25) (as mentioned in the methods section); Abbreviations: CI, Confidence Interval; and NI, Not Included.

## Data Availability

The data presented in this study are available on request from the corresponding author.
